# C-953-03. Evaluation of Triage Patterns of Burn-injured Patients Using the BEACON Model

**DOI:** 10.1093/jbcr/irag033.127

**Published:** 2026-04-06

**Authors:** Taryn E Travis, Riddhi Doshi, Katharine B Coyle, James H Holmes, IV, Rajiv Sood

**Affiliations:** The Burn Center at MedStar Washington Hospital Center, Washington, DC; IQVIA, Concord, NC; IQVIA, Falls Church, VA; Atrium Health Wake Forest Baptist Burn Center, Winston-Salem, NC; Joseph M Still Burn Centers, Carmel, Indiana

## Abstract

**Introduction:**

Accurate and consistent diagnosis of burn severity is necessary to ensure appropriate triage and transfer of burn-injured patients. We aimed to identify and describe triage patterns of burn-injured patients stratified by ABA guideline-based eligibility for transfer to burn centers (BC) and evaluate areas of potential cost savings.

**Methods:**

This retrospective, observational study evaluated IQVIA’s Hospital Charge Data Master to identify burn-injured patients who were evaluated in an emergency room (ER) or inpatient (IP) setting from 1/1/2017 to 8/31/2023. Patients were categorized by burn depth (superficial, superficial partial thickness (SPT), deep partial thickness (DPT), or full thickness (FT)) and TBSA (< 10%, 10- < 20%, 20- < 30%, 30- < 40%, 40- < 50% or > =50%). Triage patterns were evaluated based on inpatient transfer/admission to BC, admission to a non-BC, or discharge from the ER and graded against ABA guidelines for transfer to BC for admission. Triage patterns were incorporated into the Burn Efficacy And Cost Outcomes Nexus (BEACON) model to evaluate how improvement of referral patterns based on improved diagnoses achieved with multispectral imaging with artificial intelligence (AI) wound healing predictions could impact resource use and outcomes.

**Results:**

Among 28 952 adult burn-injured patients (superficial (13.5%), SPT (66.1%), DPT (1.9%), or FT (18.5%)), 21 591 (74.6%) met ABA criteria for BC transfer. Notably, among patients with either DPT and TBSA >10% or any FT burn (19.0%), 70.5% were discharged from ER (22.8%) or admitted to a non-BC (47.7%) despite meeting criteria for BC transfer. The BEACON model indicated potential reduction in LOS by 4.5 days due to faster time to decision among patients admitted to BCs and evaluated with multispectral imaging with AI. Additionally, when combined with 40% increase in appropriate BC transfers, cost savings between $6619 (2.7%) to $15 705 (6.2%) per burn-injury were estimated.

**Conclusions:**

This “real-world” data indicates that a large proportion of patients with burn-injuries presenting to an ER for initial evaluation and triage and who meet ABA transfer criteria are either discharged or transferred to a non-BC.

**Applicability of Research to Practice:**

With improved diagnosis of wound size and depth and faster time to make healing predictions, potential cost savings might be readily achievable.

**Funding for the study:**

This project is being supported in whole or in part with federal funds from the Department of Health and Human Services (DHHS); Administration for Strategic Preparedness and Response; BARDA, under contract number 75A50123C00049. The findings and conclusions have not been formally disseminated by the DHHS and should not be construed to represent any agency determination or policy.

